# DNA-aptamer-nanographene oxide as a targeted bio-theragnostic system in antimicrobial photodynamic therapy against *Porphyromonas gingivalis*

**DOI:** 10.1038/s41598-022-16310-3

**Published:** 2022-07-16

**Authors:** Maryam Pourhajibagher, Shahroo Etemad-Moghadam, Mojgan Alaeddini, Rezvaneh sadat Miri Mousavi, Abbas Bahador

**Affiliations:** 1 Dental Research Center, Dentistry Research Institute, Tehran University of Medical Sciences, Tehran, Iran; 2 Pharmaceutical Engineering Laboratory, School of Chemical Engineering, College of Engineering, University of Tehran, Tehran, Iran; 3 Fellowship in Clinical Laboratory Sciences, BioHealth Lab, Tehran, Iran

**Keywords:** Microbiology, Health care

## Abstract

The aim of this study was to design and evaluate the specificity of a targeted bio-theragnostic system based on DNA-aptamer-nanographene oxide (NGO) against *Porphyromonas gingivalis* during antimicrobial photodynamic therapy (aPDT). Following synthesis and confirmation of NGO, the binding of selected labeled DNA-aptamer to NGO was performed and its hemolytic activity, cytotoxic effect, and release times were evaluated. The specificity of DNA-aptamer-NGO to *P. gingivalis* was determined. The antimicrobial effect, anti-biofilm potency, and anti-metabolic activity of aPDT were then assessed after the determination of the bacteriostatic and bactericidal concentrations of DNA-aptamer-NGO against *P. gingivalis*. Eventually, the apoptotic effect and anti-virulence capacity of aPDT based on DNA-aptamer-NGO were investigated. The results showed that NGO with a flaky, scale-like, and layered structure in non-cytotoxic DNA-aptamer-NGO has a continuous release in the weak-acid environment within a period of 240 h. The binding specificity of DNA-aptamer-NGO to *P. gingivalis* was confirmed by flow cytometry. When irradiated, non-hemolytic DNA-aptamer-NGO were photoactivated, generated ROS, and led to a significant decrease in the cell viability of *P. gingivalis* (*P* < 0.05). Also, the data indicated that DNA-aptamer-NGO-mediated aPDT led to a remarkable reduction of biofilms and metabolic activity of *P. gingivalis* compared to the control group (*P* < 0.05). In addition, the number of apoptotic cells increased slightly (*P* > 0.05) and the expression level of genes involved in bacterial biofilm formation and response to oxidative stress changed significantly after exposure to aPDT. It is concluded that aPDT using DNA-aptamer-NGO as a targeted bio-theragnostic system is a promising approach to detect and eliminate *P. gingivalis* as one of the main bacteria involved in periodontitis in periopathogenic complex in real-time and in situ.

## Introduction

Periodontitis is a microbial biofilm-mediated chronic oral inflammatory disease of the supporting tissues of the teeth that progresses with root resorption, and subsequent loss of the teeth^1,2^. Today, surgical and non-surgical treatments are performed for periodontitis. Pharmacological intervention and surgery are considered the gold standard therapeutic protocol for advanced periodontitis, which may be associated with a high level of displeasure in the patient^3^. Although non-surgical treatment can improve important clinical parameters, microbial pathogens are not significantly reduced and this approach is not also effective in moderate to severe periodontal lesions^4–6^. Periodontitis is a polymicrobial disease and *Porphyromonas gingivalis*, as one of the most common bacterial causes, forms a microbial biofilm in the periodontium and on the teeth through the expression of various virulence factors, which eventually leads to treatment failure^7^.

*P. gingivalis* produces fimbrillin (*fimA*) and arginine protease (*rgpA*) genes that are involved in several functions including adherence to host cells, formation of microbial biofilm, inhibition of host defenses, and damage to host cells^8,9^. Additionally, *P. gingivalis* has a sophisticated regulation system which is known as the oxidative stress response gene (*oxyR*) that protects the bacteria against harsh environments^10^. Systemic treatment with metronidazole or tetracycline may reduce the load of *P. gingivalis* but not be able to eradicate the organism^11^. Therefore, new therapeutic strategies such as antimicrobial photodynamic therapy (aPDT) can be used to kill microorganisms. aPDT as a non–invasive therapeutic modality for the treatment of various infections, is defined as an oxygen-dependent photochemical reaction that occurs upon irradiation with light of specific wavelength-mediated activation of a photosensitizer leading to the generation of cytotoxic reactive oxygen species (ROS) such as singlet oxygen (^1^O_2_), hydroxyl radical (•OH), and superoxide radical anion (O_2_^•−^)^12–17^.

Photosensitizers used in aPDT are classified into two major groups based on their origin; natural (curcumin, Hypericin, Flavin derivatives, and Tetra-pyrroles) and synthetic (Phenothiaziniums including methylene blue, toluidine blue, and Rose Bengal). Recently the application of photosensitizers in nanoscales had a great impact on aPDT efficacy^18^. Nanographene oxide (NGO) as a carbon-based nanomaterial has emerged rapidly as a promising material for use in drug delivery, biomolecule recognition, bioassays, and aPDT due to its excellent surface functionality, amphiphilicity, aqueous appearance, fluorescence quenching ability, and surface-enhanced Raman’s scattering property^19^.

GO is an oxidized form of graphene in the single-atomic layer, which is abundant and cheap. It is commonly found in powder form and its process is considered easy since it is dispersible in numerous solvents including water. The supremacy of GO on the nanoscale (i.e., NGO) is essentially in its own intrinsic physical and chemical structure, which confers unusual physical properties and extraordinary chemical versatility. The great abundance of oxygen functional groups on the huge carbon structure in the surface area of NGO makes possible its relatively easy functionalization with biological structures or organic molecules via hydrophobic, π- πstacking, electrostatic, and hydrogen bonding interactions.

The fluorescence study of GO using steady-state and time-resolved spectroscopic techniques revealed the excitation wavelength-dependent fluorescence and the multiexponential fluorescence decay kinetics at the emission wavelengths (λem) = 500–800 nm. It is demonstrated that the excitation spectrum of GO broadens with the λem and there was no sharp absorption peak. All functionalized groups C=O, C–O, and O=C–OH in NGO are involved in the fluorescence of GO^20^.

**Figure 1:**
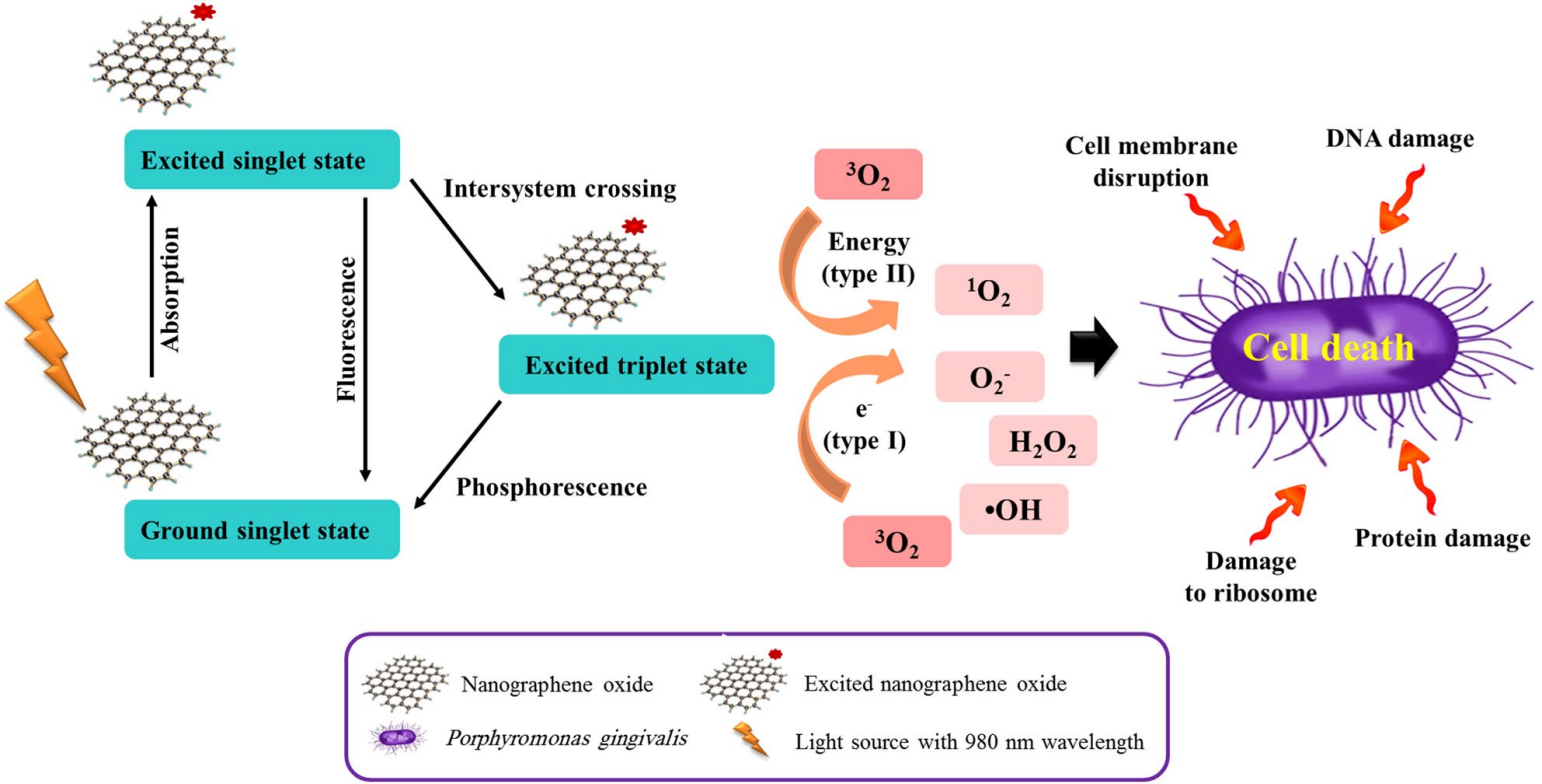
Schematic illustration of antimicrobial photodynamic therapy mechanism using nanographene oxide (NGO).

The killing actions of aPDT against microorganisms are immediate and independent of their pattern of antimicrobial resistance, while most antibiotics take hours or days to work. aPDT has a low likelihood of selection of drug-resistant microbial strains due to its multi-target mechanism of antimicrobial actions. aPDT displays several advantages such as selective targeting, non-invasiveness, low cytotoxicity, high spatiotemporal precision, no drug resistance, and synergistic effect over conventional therapeutic interventions^21^. It has been found that aPDT is effective against all types of microorganisms, some of them are more susceptible than others. Produced ROS can cause damage to DNA and external cell structures, including the cytoplasmic and outer membrane in Gram-negative bacteria, and, the cytoplasmic membrane in Gram-positive bacteria which lead to the death of the microorganisms^21^ (Fig. 1). It has revealed that ^1^O_2_ is the major and the key ROS produced following irradiation of NGO^12,13^, which are harmful to cell membrane integrity, causing irreparable biological damage, and microbial cell death without damaging the surrounding tissue^14–17^.

Biofunctionalization of NGO with oligonucleotides has been reported and NGO embedded with DNA is used to form different nanoparticles and other Biosystems^22^. Single-stranded short oligonucleotides (DNA or RNA) called “aptamers” are third-generation molecular probes that can tightly bind to their target molecule with high affinity^23,24^.

Aptamers are selected in vitro from a synthesized random library using systematic evolution of ligands by exponential enrichment (SELEX)^25,26^. In cell-SELEX, the whole of the cell has been shown to be targeted. Aptamers can be considered cost-effective diagnostic methods that overcome the side effects of therapeutic agents and drug resistance of microbial pathogens. They can bind specifically to the surface factors of microorganisms, block the function of target proteins, and therefore be used as a basis for developing new therapies for infections^22^. Therefore, the use of aptamers in drug delivery systems has been considered due to their properties.

Accordingly, in the present study, the use of aptamer in addition to the specific identification of *P. gingivalis* as a photosensitizer carrier (NGO) is suggested. In this study, the synthesized NGO was first bonded to a DNA-aptamer that has been selected against *P. gingivals*, and its physical stability, hemolytic effect, and cytotoxicity potential were evaluated. Next, the specificity of DNA-aptamer-NGO to *P. gingivalis* was monitored. After evaluation of endogenous ROS production, the antimicrobial, anti-biofilm, and anti-metabolic activities of aPDT based on DNA-aptamer-NGO against *P. gingivalis* were assessed. Finally, the apoptotic effects of aPDT using DNA-aptamer-NGO in human gingival fibroblast cells and the expression of genes involved in *P. gingivalis* pathogenesis were evaluated. The hypothesis in this study is that aptamer with specific binding to *P. gingivalis* firstly the other beneficial microorganisms are not affected by targeted aPDT, and secondly increases the antimicrobial and anti-biofilm effects of NGO-based aPDT without induction of apoptosis in normal human gingival fibroblast (HGF) cells.

## Materials and methods

### Synthesis of NGO

The synthesis of NGO oxide was performed using modified Hummer’s method^27^ in following manner: 3 g of graphite powder was added to a mixture of 69 mL sulfuric acid (H_2_SO_4_, 98%) and sodium nitrate (NaNO_3_, 1.5 g) in an ice bath for 2 h. 9 g of powdered potassium permanganate (KMnO4, 99%) was then slowly added to the mixture under continuous stirring at a temperature lower than 14 °C. The ice bath was removed after 2 h of stirring and the mixture was stirred until it turned dark green. 100 mL of distilled water was added slowly to the reaction mixture and the solution was then sonicated for 30 min. After that, 2 mL of 30% H_2_O_2_ was added dropwise to the suspension causing the color to turn yellow, indicating the termination of the reaction. Finally, the mixture was centrifuged and rinsed with 10% HCl solution to remove the remaining metal ions. The GO powder was collected through filtration and dried under a vacuum.

### Characterization of NGO

The surface morphology of the prepared NGO was evaluated with field emission scanning electron microscopy (FESEM, ZEISS, Germany). The particle size and zeta potential of nanoparticles were characterized by a MALVERN Zetasizer Ver. 6.01 (Malvern Instruments, UK). Fourier-transform infrared (FTIR) analysis was performed using a spectrum of two spectrophotometers (45° ZnSe crystal, PerkinElmer Inc., US) in the range of 500–4000 cm^−1^.

### Bacterial strain and culture condition

*P. gingivalis* IR-TUMS/BPG5 (Accession number in Genbank: KX108929.1) was cultured on sheep blood agar plates containing brucella agar (Merck, Darmstadt, Germany) supplemented with 0.5% defibrinated sheep blood, 0.6% yeast extract, 5 mg/L hemin, and 1 mg/L menadione (all purchased from Sigma-Aldrich, Germany) at 37 °C in an anaerobic atmosphere composed of 10% H_2_, 5% CO_2_, and 85% N_2_ at 37 °C for 7 days.

### Binding of DNA-aptamer to NGO

**Figure 2:**
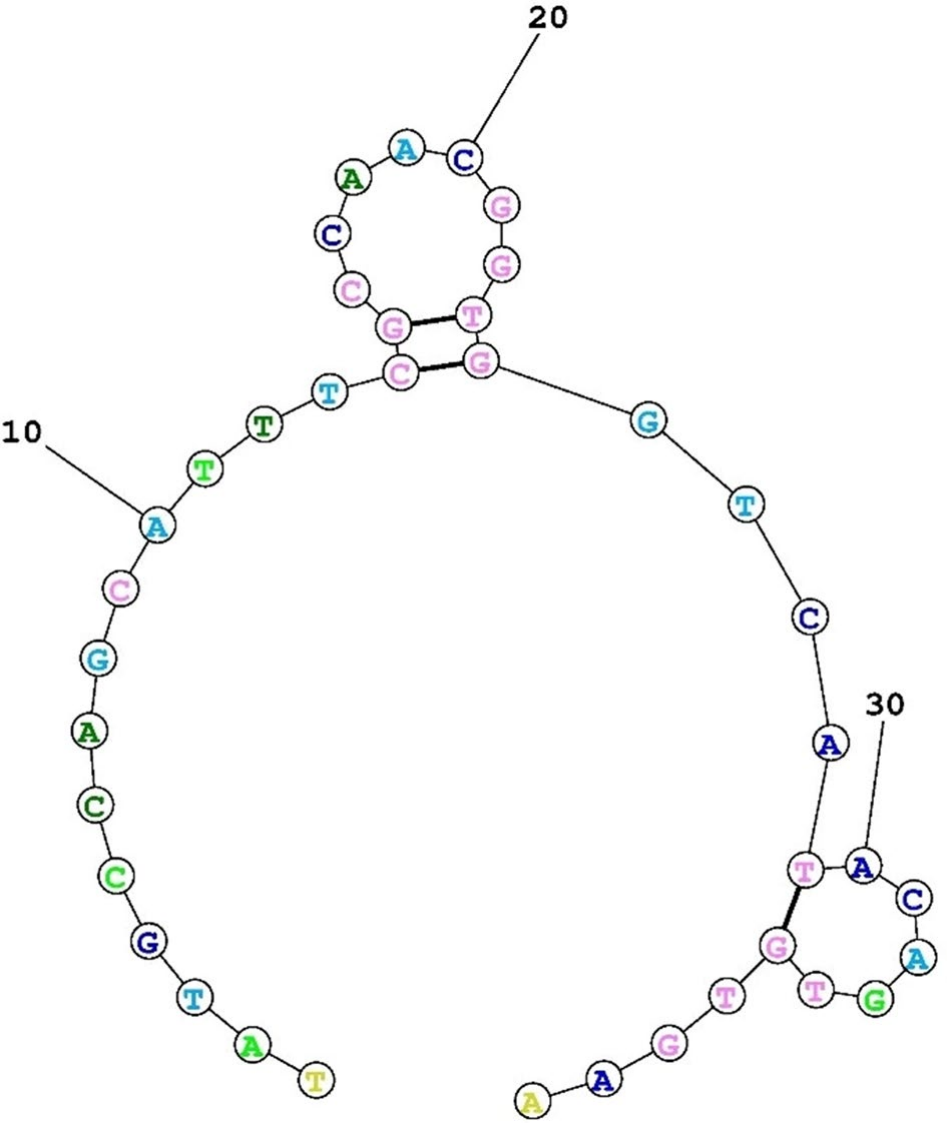
The secondary structure of the deoxyribonucleic acid-aptamer specific to *P. gingivalis*.

Nucleotide sequences of aptamer specific to *P. gingivalis* were selected based on Park et al. study^28^. The sequence of the aptamer was optimized and modified by fluorescein amidites (FAM) based on the previous study^29^. The structure of the aptamer is shown in Fig. 2. The sequence of the FAM-labeled DNA-aptamer was 5’-TATGCCAGCATTTCGCCAACGGTGGTCATACAGTGTGAA-3’. The binding of labeled DNA-aptamer to NGO was performed by the modified Lin et al. method^30^. Briefly, the oligonucleotide sequence was exposed to 40 μg/mL of NGO and allowed to react. After 2 h of reaction, 1% NaCl solution (1 M) was added and the solution was maintained at 4 °C for 30 min. The mixture was then incubated at 25, 45, 60, and 90 °C for 1 h. The resulting mixture was centrifuged at 16,000 rpm for 45 min and the residues were resuspended with phosphate-buffered saline (PBS; pH ~ 7.4). After three centrifugations and washing cycles, the aptamer-NGO was obtained. Afterward, the fluorescence intensities of the mixtures were recorded using a spectrophotometer. The emission spectra and the excitation wavelength were measured at 510 and 480 nm, respectively.

### Evaluation of binding of DNA-aptamer-NGO composite to the *P. gingivalis*

To direct evidence of the binding of DNA-aptamer-NGO composite material to the *P. gingivalis* as the target, we compared the change in cell size after exposure of *P. gingivalis* cells to the DNA-aptamer-NGO composites with the control group (untreated *P. gingivalis*) using fluorescence intensity assay and FESEM. In brief, fresh supplemented BHI bacterial cultures, in the logarithmic growth phase (9–12 h old; adjusted to a concentration of 10^8^ CFU/mL), were treated with the composites (250 nM) for10 min. Upon centrifugation at 6000 rpm for 8 min, the bacterial cells were collected and washed with PBS (pH 7.5) twice to remove unattached DNA-aptamer-NGO composite material and then resuspended in sterile distilled water. The bacterial suspension was filtered through a 0.2 μm polycarbonate filter to reassure the removal of unattached DNA-aptamer-NGO composite material and the filters containing bacterial cells as specimens were used for fluorescence intensity assay and FESEM. In the fluorescence intensity assay the restoration of fluorescent intensity to the samples was measured using a fluorescence spectrophotometer using excitation and emission wavelengths of 492 and 532 nm, respectively. In the FESEM, samples were fixed in a glutaraldehyde solution (2.5% glutaraldehyde in 0.2 M sodium cacodylate/hydrochloric acid buffer, pH 7.5) as described in our previous study^31^. The specimens were rinsed with the sodium cacodylate/hydrochloric acid buffer two times, followed by post fixing with 1% aqueous osmium tetroxide and dehydrated with graded ethanol solutions (50% for 30 min, 75%, 85%, and 95% each for 10 min, and 100% for 10 min). After critical point drying to remove ethanol completely, the specimens were mounted onto a stub, coated by gold sputter, and examined by FESEM as previously described^31^. To verify the detection of the *P. gingivalis* as the target bacterium, the fluorescent increment depending on an increment of cell numbers (10^2^, 10^4^, and 10^6^ CFU/mL) was assessed using DNA-aptamer-NGO composites (250 nM) based on the fluorescence intensity assay used in the evaluation of the binding of DNA-aptamer-NGO composite to the *P. gingivalis*. The intensities of FAM-DNA aptamer-NGOs (250 nM, 500 nM, and 1000 nM without any bacterial cells) also were measured according to the fluorescence intensity technique.

### Evaluation of in vitro effect of pH on aptamer binding

The effects of pH value on aptamer binding were evaluated as described previously^32^. Briefly, following the preparation of the DNA-aptamer-NGO composite, it was washed using buffer A (1 Mm MgCl_2_, 150 mM NaCl, 25 mM HEPES, pH 7.6) and centrifugation of the mixture at 15,000 rpm for 20 min to remove unbound aptamers. The DNA-aptamer-NGO composite pellet was then dissolved in bacterial (*P. gingivalis*) suspension (10^6^ CFU/mL). After that, the ionic strength of the suspension was adjusted to high using buffer A (150 mM NaCl, 25 mM HEPES, pH 7.6). Fluorescence intensities of the suspension were then measured following acidification and alkalization by incubating with citrate buffer (1 M; pH 5.5) and sodium bicarbonate (NaHCO_3_; 1 M; pH 8.5), respectively using a UV–Vis spectrophotometer at 430 nm.

### Hemolysis assays

The biocompatibility of DNA-aptamer-NGO composite based on ASTM standard E2524-08 (Standard Test Method for Analysis of Hemolytic Properties of Nanoparticles)^33^ was determined using measurement of the hemolytic activity of the composite, as described by Isnansetyo and Kamei^34^. Briefly, a human whole blood sample obtained from returned unused blood bags in the blood bank (Iranian Blood Transfusion Organization) was centrifuged at 900 g for 2 min and then washed three times with phosphate-buffered saline (PBS; pH ~ 7.4) and diluted in PBS (330 μL of human whole blood per 10 ml of PBS). 0.2 mL of the diluted human whole blood sample was mixed with 0.8 mL of DNA-aptamer-NGO at the different concentrations (250, 500, and 1000 nM). The mixtures were incubated at a 37 °C water bath for 1 h. After the incubation, the samples were centrifuged at 800 g for 15 min and the absorbance of the supernatant was analyzed by a UV–Vis spectrophotometer at 540 nm.

PBS (pH ~ 7.4) was chosen as a negative control since it is compatible with blood cells. Distilled water (pH ~ 7.4) was applied as a positive control due to its high hemolytic activity. Hemolysis assays were done in triplicate. The hemolytic activity results of DNA-aptamer-NGO in different concentrations as the samples expressed as percentage hemolysis with respect to negative and positive controls as follows:

$$\mathrm{Hemolysis rate }(\mathrm{\%})=\frac{{\mathrm{D}}_{\mathrm{sample}}-{\mathrm{D}}_{\mathrm{negative\,\,\, control}}}{{\mathrm{D}}_{\mathrm{positive\,\,\, control}}-{\mathrm{D}}_{\mathrm{negative\,\,\, control}}} \times 100$$

### Cytotoxicity effect of DNA-aptamer-NGO on human gingival fibroblast cell

Primary human gingival fibroblast (HGF) cells obtained from the Iranian Biological Resource Center (CELL NO. IBRC C10459) were seeded at a density of 1 × 10^5^ cells/well in a 96-well microtiter plate containing Dulbecco’s modified Eagle medium (Gibco, Grand Island, NY) supplemented with 10% fetal bovine serum (Gibco), 2% l-glutamine, 100 U/mL penicillin, and 100 g/mL streptomycin (all purchased from Sigma-Aldrich, Germany). The microtiter plate was incubated at 37 °C in a humidified atmosphere of 5% CO_2_ in the air for 24 h. After cells washing with PBS DNA-aptamer-NGO at the different concentrations (250, 500, and 1000 nM) were added to triplicate wells and kept for 24 h. After re-washing, the viability of HGF cells was assessed using MTT [3-(4,5-dimethylthiazol-2-yl)-2,5-diphenylte-trazolium bromide] kit according to the manufacturer’s instructions. Finally, the optical density (OD) at 570 nm was measured by a microtiter plate.

### Monitoring of specificity of DNA-aptamer-NGO to *P. gingivalis*

The specificity of binding of the FAM-labeled aptamer-NGO to *P. gingivalis* was assessed using flow cytometry. Briefly, 250 nM of FAM-labeled aptamer was incubated with 10^6^ CFU/mL of *P. gingivalis* as the target cell and different bacteria such as *Streptococcus mutans*, *Enterococcus faecalis*, and *Aggregatibacter actinomycetemcomitans* as the non-target cells at 37 °C for 2 h. After washing with PBS three times, the bacteria cells were resuspended in PBS (300 μL). The fluorescence intensity of bacteria was measured by flow cytometry based on the previous study^35^.

### Determination of bacteriostatic and bactericidal concentrations of DNA-aptamer-NGO against *P. gingivalis*

To determine the minimum inhibitory concentration (MIC) and minimum bactericidal concentration (MBC) of DNA-aptamer-NGO as the bacteriostatic and bactericidal concentrations, respectively, we followed the procedure by acting according to our previous study^36^. In brief, after adding 100 µL of BHI broth supplemented with hemin and menadione to each well of a 96-well microtiter plate, 100 µL of 2000 nM DNA-aptamer-NGO was added to the well in column one and diluted twofold step-wise to column ten. Then, 100 µL/well of bacterial suspension with a concentration of 1.0 × 10^6^ CFU/mL was transferred to each well. The microtiter plate was incubated under anaerobic conditions at 37 °C for 24 h. According to Clinical and Laboratory Standards Institute (CLSI) guidelines^37^, MIC was determined as the lowest concentration of DNA-aptamer-NGO that completely inhibits visible bacterial growth, and MBC was determined by sub-culturing the test dilution on BHI agar plates that caused at least 99.999% killing of the initial inoculum.

### Light source

In this study, a DenLase, Diode Laser Therapy System (Daheng Group Inc., China) equipped with 980 nm wavelength, a power of 1 W, a continuous-wave operation, and fiber of 400 μm was used as a light source^38^.

### Evaluation of endogenous ROS production

In this study, endogenous ROS generation was quantified by fluorescence spectroscopy using 2′,7′-dichlorofluorescein diacetate (H_2_DCF-DA) according to the previous study^39^. Overnight culture of *P. gingivalis* was centrifuged (10,000 rpm for 15 min) and the pellets were washed and resuspended in PBS to achieve the cell density of 10^8^ CFU/mL followed by incubation with 10 μM DCFH-DA. After 10 min of incubation, the bacterial cells were treated with 1/2 × and 1/4 × MIC of DNA-aptamer-NGO and exposed to the diode laser light for 1 min. In only light and only DNA-aptamer-NGO treated groups, the cells were exposed to the laser light without DNA-aptamer-NGO, and DNA-aptamer-NGO without laser light, respectively, while the control group (only bacterial cells) was left untreated. Thereafter, the fluorescence intensity produced from DCFH-DA was measured by a fluorescence spectrophotometer at an excitation wavelength of 488 nm and an emission wavelength of 535 nm.

### Antimicrobial effect of aPDT based on DNA-aptamer-NGO against *P. gingivalis* in planktonic form

The antimicrobial effect of aPDT against *P. gingivalis* was determined as described by Pourhajibagher et al.^40^. Briefly, 100 µL of 1/2 × and 1/4 × MIC of DNA-aptamer-NGO was added to the wells after the addition of enriched BHI broth (100 µL) to each well of a 96-well microtiter plate. 100 µL/well of *P. gingivalis* suspension at the concentration of 1.0 × 10^6^ CFU/mL was then added to each well. The suspension was incubated under dark, anaerobic conditions for 5 min and immediately exposed to the diode laser irradiation. Eventually, 10 µL of each well was spread onto the enriched BHI agar and the plates were incubated at 37 °C for 48 h in an anaerobic condition to determine *P. gingivalis* Log_10_ CFU/mL.

### Anti-biofilm effect of aPDT based on DNA-aptamer-NGO against *P. gingivalis*

*P. gingivalis* biofilm was formed in a 96-well microtiter plate according to the previous study^41^. Briefly, 200 μL of *P. gingivalis* suspension at a final concentration of 1.5 × 10^8^ CFUs/mL was added to each well of a 96-well microtiter plate and incubated at 37 °C for 48 h to allow biofilm formation. Prior to the treatment, the remaining non-adherent bacteria were removed by washing with PBS. After that, the preformed biofilm was incubated with 1/2 × and 1/4 × MBC of DNA-aptamer-NGO in the dark for 5 min and then treated with the diode laser. Biofilms in all the wells were subsequently stained using 100 μL of 0.1% crystal violet. After 15 min of incubation at room temperature, the wells were washed with PBS and the unbound dye was removed from the cells with 100 μL of 95% ethanol. After two rinses with PBS and drying in the air, 150 μL of 33% acetic acid was added to the wells and each sample’s absorbance was determined at 570 nm using a microplate reader.

### Assessment of anti-metabolic activity of aPDT based on DNA-aptamer-NGO

The metabolic activity of *P. gingivalis* treated aPDT using 1/2 × and 1/4 × MIC of DNA-aptamer-NGO was determined using the XTT (2,3-bis [2-methyloxy-4-nitro-5-sulfophenyl]-2H-tetrazolium-5-carboxanilide) reduction assay, as Coraça-Hubér et al.^42^ study. After each treatment, the contents of the wells were collected and the microbial suspensions were centrifuged at 2000 rpm for 10 min. Afterward, the microbial cell sediments were dissolved in XTT-menadione-PBS solution (150 μL) and incubated at 37 °C. After 3 h, 100 μL of the solution was transferred to a new microtiter plate and the OD measurements were performed at 492 nm using a microplate reader.

### Estimation of the apoptotic effects of aPDT based on DNA-aptamer-NGO by flow cytometry

1 × 10^6^ cells/mL of HGF cells were cultured in a 96-well microtiter plate and incubated under the conditions mentioned above. 24 h later, DNA-aptamer-NGO at concentrations of 1/2 × and 1/4 × MIC were added to the culture medium. After six h, the cells in the DNA-aptamer-NGO-aPDT group were exposed to the diode laser light for 1 min. The cells were then washed with ice-cold PBS. The washed cells were re-suspended in 1 × binding buffer, and then 5 μL Annexin V-FITC was added followed by 5 μL propidium iodide (PI). The cells were incubated at room temperature in the dark for 15 min, and the percent of apoptotic cells was then determined by flow cytometry.

### Quantification of the expression of genes via reverse transcription-quantitative real-time PCR (RT-qPCR)

Immediately after treatment of *P. gingivalis* by aPDT based on 1/2 × and 1/4 × MIC of DNA-aptamer-NGO, the cells were pelleted and total RNA was extracted using the super RNA extraction Kit (AnaCell, Iran) in accordance with the manufacturer’s instructions. The extracted RNAs were first treated with RNase-free DNase I treatment (Thermo Scientific GmbH, Germany) to eliminate the genomic DNAs, and cDNAs were then synthesized using random hexamer primed reactions using a Revert Aid First Strand cDNA Synthesis Kit (Thermo Fisher Scientific, US), according to the manufacturer’s protocol. The gene-specific primers were designed according to our previous studies^43–45^ and listed in Table 1.

**Table 1:** Primer sequences used in this study.

Genes		Sequences (5′–3′)^a^	Amplicon Size (bp)	References
*fimA*	F	CGGAACGAATAACCCAGAGA	88	35
	R	CTGACCAACGAGAACCCACT		
*rpgA*	F	CGGTGAGCGTCAAGTTCGTT	120	36
	R	GTGCTATCGCTACGCTCTC		
*oxyR*	F	CCACAACTGACCGTAGAGCA	188	37
	R	CCTGTCTGCAACTTGTGCAT		
*16S rRNA*	F	TGACACTGAAGCACGAAAGC	166	36
	R	TCCTTGAGTTTCACCGTTGC		

F, forward primer; R, reverse primer; and bp, base pair.

^a^Nucleotides.

RT-qPCR was performed using Line-GeneK Real-Time PCR Detection System and Software (Bioer Technology, Hangzhou, China) under the cycling conditions included 95 °C for 5 min, followed by 40 cycles of 95 °C for 15 s, 60 °C for 10 s, and 72 °C for 10 s. The relative fold change was calculated using Livak and Schmittgen (2^−ΔΔCT^) method^46^.

### Statistical analysis

All assays were set up in triplicate, and the results were expressed as mean values ± standard deviations (mean ± SD). Statistical significance was determined by two-way analysis of variance (ANOVA) and Tukeys’ test in SPSS statistical software version 23. All experiments were performed in at least triplicate. P-values of less than 0.05 were considered to be statistically significant.

### Ethics approval and consent to participate

The study was approved by the Ethics Committee of Tehran University of Medical Sciences (Application No. IR.TUMS.DENTISTRY.REC.1399.253), and all methods were carried out in accordance with relevant guidelines and regulations.

## Results

### Confirmation of NGO synthesis

**Figure 3:**
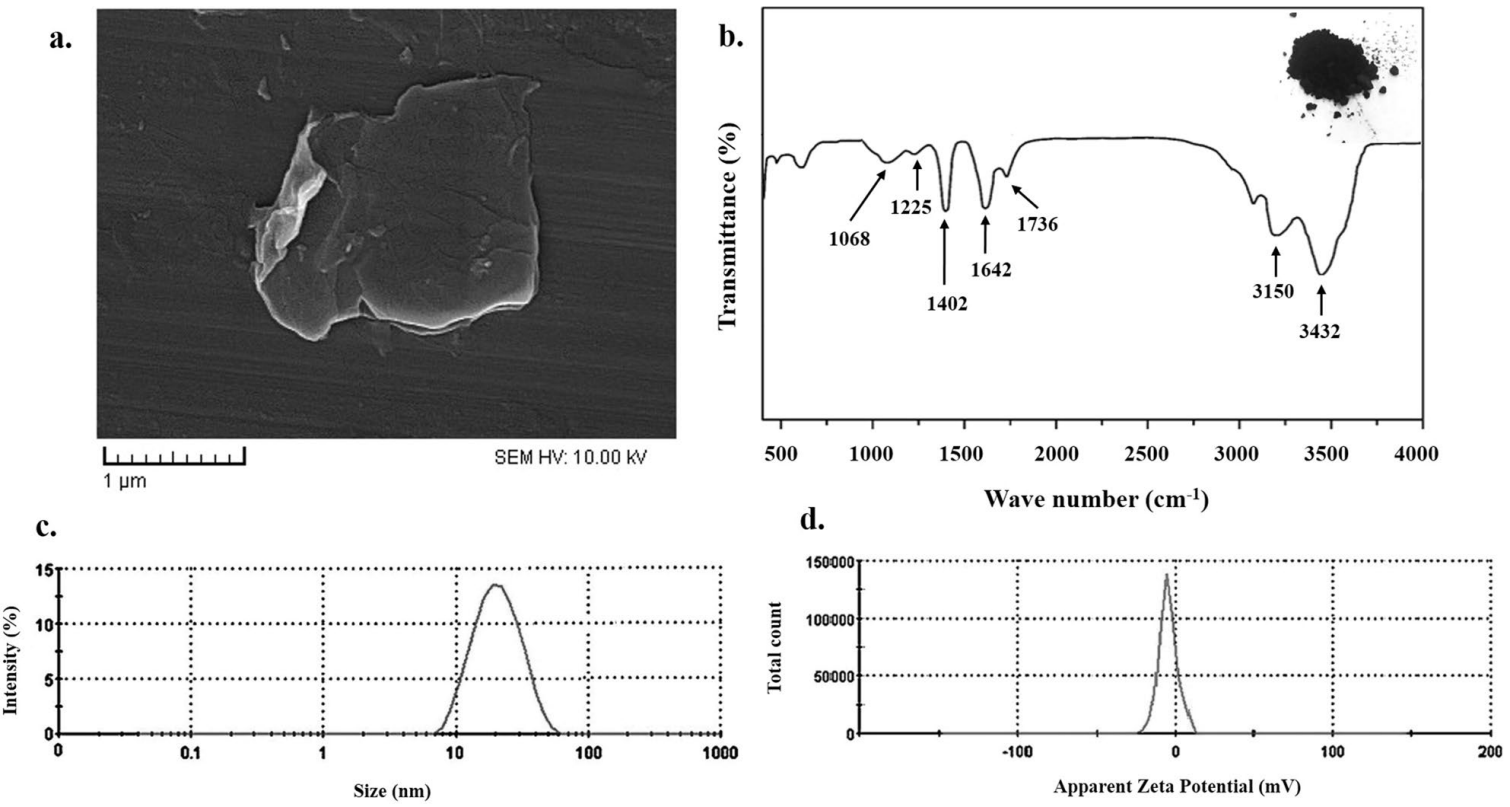
Characterization of synthesized NGO: (**a**) Micrograph of field emission scanning electron microscopy (FESEM); NGO with transparent rippled silk-like waves or a flaky, scale-like, layered structure, and wrinkled edge. (**b**) Fourier-transform infrared (FTIR) in the range of 500–4000 cm^−1^, the peaks at 1402 cm^−1^ and 3432 cm^−1^ indicate the vibrational bands of O–H. C–O stretching, C=C stretching, and C=O stretching are observed at 1225 cm^−1^, 1642 cm^−1^, and 1736 cm^−1^, respectively. (**c**) Average particle size distribution of NGO is 21.3 ± 3.2 nm. (**d**) The zeta potential of NGO is −17.1 mV.

The FESEM image of NGO in Fig. 3a exhibits transparent rippled silk-like waves or a flaky, scale-like, layered structure, and wrinkled edge. Figure 3b shows the FTIR spectra of the NGO in the range of 500–4000 cm^−1^, in which the peaks at 1402 cm^−1^ and 3432 cm^−1^ indicate the vibrational bands of O–H. C–O stretching, C=C stretching, and C=O stretching are observed at 1225 cm^−1^, 1642 cm^−1^, and 1736 cm^−1^, respectively. The peak at 1068 cm^−1^ is attributed to a stretching vibration from the C–O–C bonds of epoxy or alkoxy groups. According to the results in Fig. 3c and d, the average size and the zeta potential of NGO were 21.3 ± 3.2 nm and −17.1 mV, respectively.

### DNA-aptamer-NGO fluorescent for detection of *P. gingivalis*

**Figure 4:**
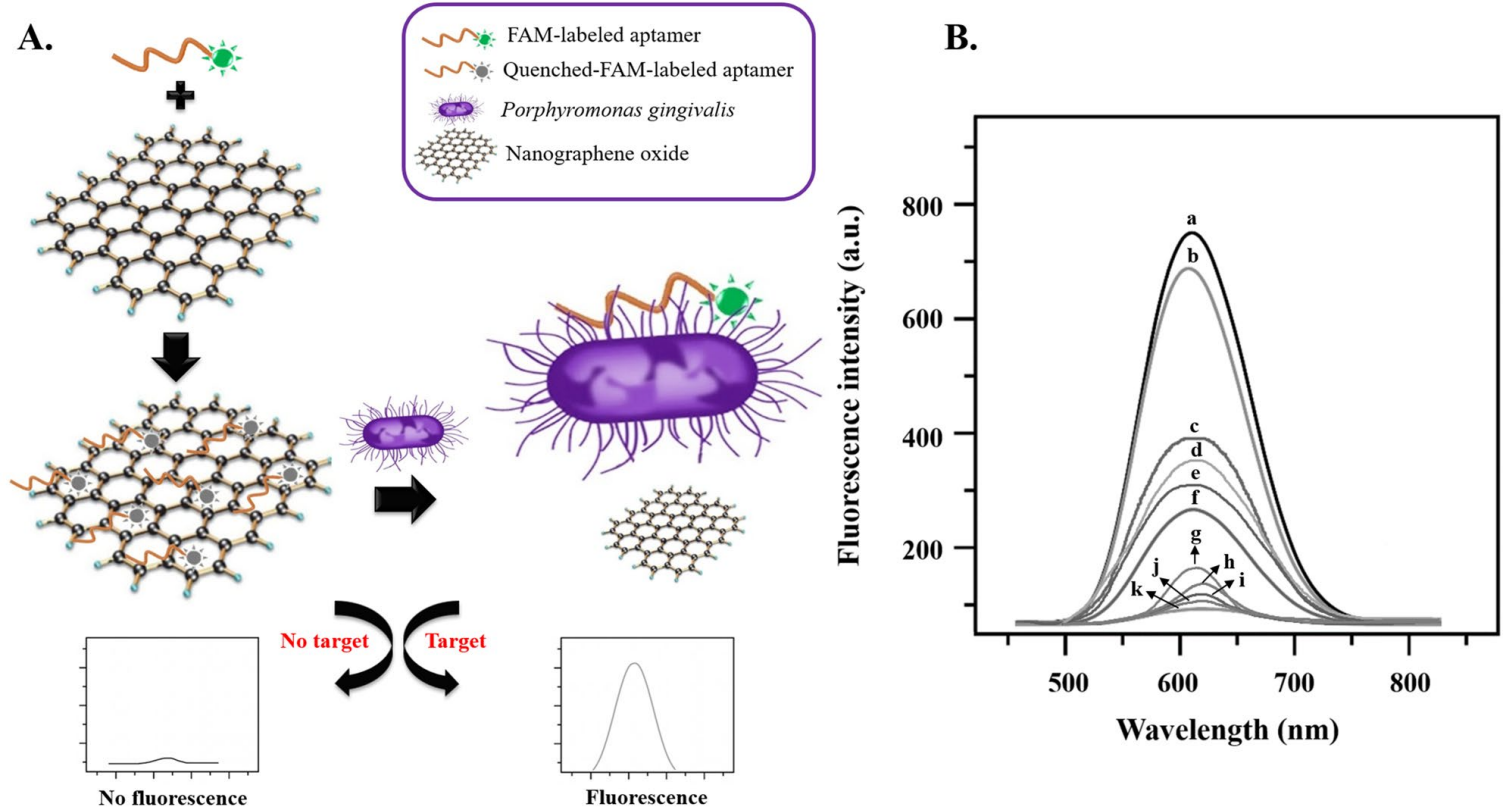
(**A**) Schematic illustration of DNA-aptamer-NGO fluorescent for detection of *P. gingivalis*. (**B**) Fluorescence emission spectra of FAM-DNA-aptamer and *P. gingivalis* at different conditions: (a) FAM-DNA-aptamer, (b) FAM-DNA-aptamer-*P. gingivalis* (10^6^ CFU/mL), (c) FAM-DNA-aptamer-NGO (250 nM)- *P. gingivalis* (10^6^ CFU/mL), (d) FAM-DNA-aptamer-NGO (250 nM)- *P. gingivalis* (10^4^ CFU/mL), (e) FAM-DNA-aptamer-NGO (250 nM) *P. gingivalis* (10^2^ CFU/mL), (f) FAM-DNA-aptamer-NGO (500 nM)-*P. gingivalis* (10^6^ CFU/mL), (g) FAM-DNA-aptamer-NGO (1000 nM)-*P. gingivalis* (10^6^ CFU/mL), (h) FAM-DNA aptamer-NGOs at concentrations of 250 nM, without any bacterial cells, (i) FAM-DNA aptamer-NGOs at concentrations of 500 nM without any bacterial cells, (j) FAM-DNA aptamer-NGOs at concentrations of 1000 nM without any bacterial cells, and (k) *P. gingivalis* with a negligible fluorescent intensity.

In the current study, NGO and FAM-DNA-aptamer were used to design a fluorescent bio-theragnostic agent to detect *P. gingivalis* cells. According to Fig. 4A, in the absence of *P. gingivalis*, FAM-DNA-aptamer is adsorbed onto the surface of NGO via weak binding force by π-π stacking, and NGO quenches the fluorescence of FAM. In the presence of *P. gingivalis*, the FAM-DNA-aptamers fall off from the NGO surface and bind to the *P. gingivalis*, causing fluorescence restoration. As shown in Fig. 4B, the fluorescence spectrum of the FAM-DNA-aptamer presents strong fluorescence intensity (curve a). The fluorescence intensity of FAM-DNA-aptamer without NGO has no obvious change when *P. gingivalis* was added (curve b). After mixing the NGO (250 nM), the fluorescence intensity was reduced (curve c), indicating that NGO quenched the fluorescence and the quenching of the fluorescence is NGO-concentration depended manner. Under the same conditions, the higher quenching of the fluorescence is due to the increase in the concentration of NGO (curves f and g stand for 500 and 1000 nM of NGO, respectively). As shown in Fig. 4 (curves c, d, and e), in an increment of cell numbers of *P. gingivalis* (10^6^, 10^4^, and 10^2^ CFU/mL, respectively) using DNA-aptamer-NGO composites (250 nM), more FAM-DNA-aptamers are separated from the NGO surface and attached to the bacterial cell surface, resulting in increasing the intensity of the fluorescence intensity. Due to the absence of *P. gingivalis*, FAM-DNA-aptamers were adsorbed onto the surface of NGO, and NGO quenched the fluorescence of FAM. The intensities of FAM-DNA aptamer-NGOs at concentrations of 250 nM, 500 nM, and 1000 nM without any bacterial cells were shown in curves h, I, and j, respectively. As revealed in these curves, with increasing NGOs concentration, fewer fluorescence intensities were observed.

### Confirmation of binding of DNA-aptamer to the NGO using atomic force microscopy (AFM)

**Figure 5:**
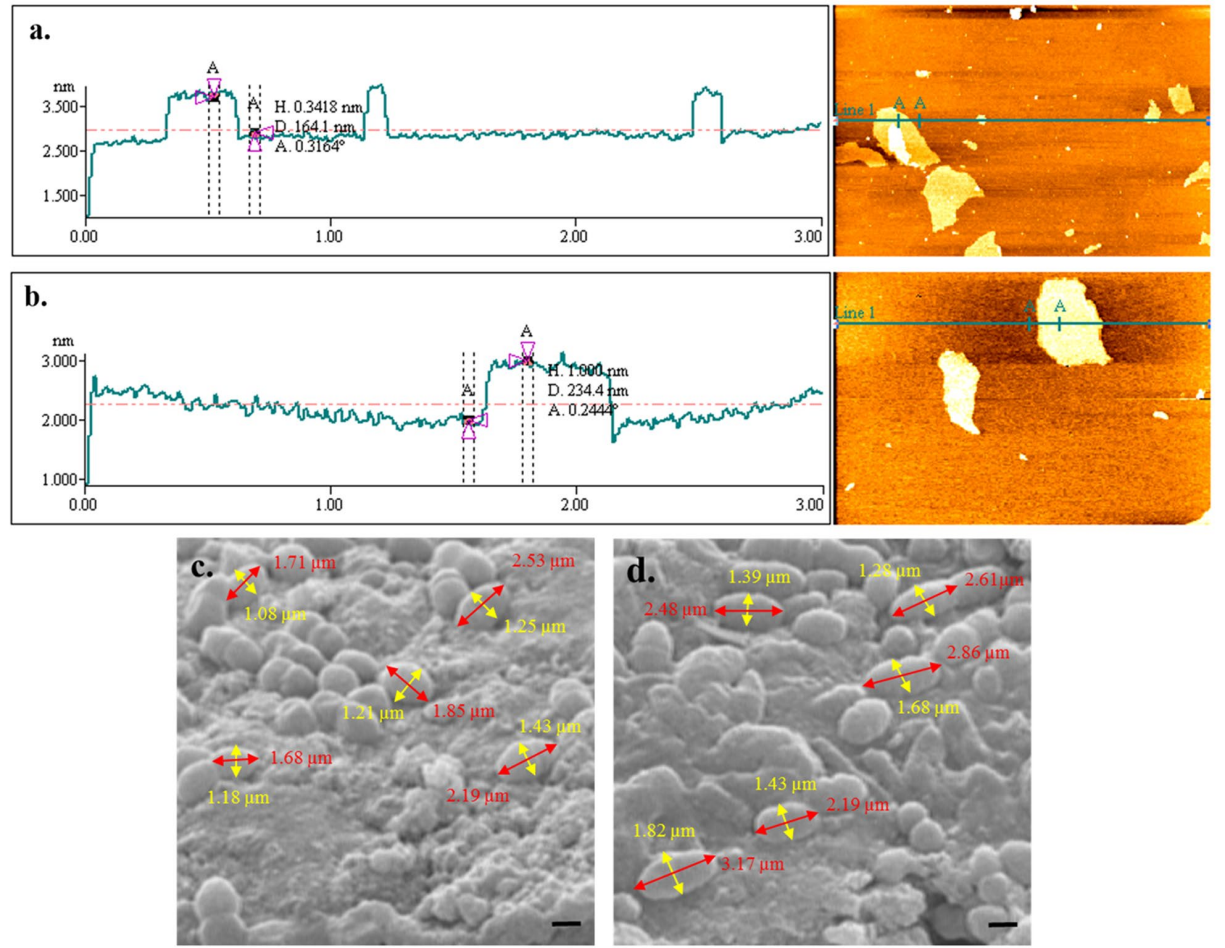
Images of binding of DNA-aptamer to the NGO (**a** and **b**) and binding of DNA-aptamer-NGO composite material to the *P. gingivalis* using AFM and SEM, respectively. NGO sheet thickness: 0.34 nm (**a**), DNA-aptamer-NGO thickness:1.00 nm (**b**), untreated *P. gingivalis* cells (length, 1.99 ± 0.36 μm; diameter, 1.23 ± 0.12 μm) with intact surfaces (**c**), and DNA-aptamer-NGO composite treated *P. gingivalis* (length, 2.66 ± 0.37 μm; diameter, 1.52 ± 0.22 μm) (**d**) with increased mean size of the bacteria (Scale bar = 500 nm).

The binding of DNA-aptamer to the NGO and formation of DNA-aptamer-NGO composite was verified by AFM. As shown in Fig. 5a, the NGO sheet thickness is 0.34 nm. However, DNA-aptamer-NGO with a thickness of 1.00 nm showed that FAM-aptamer has been absorbed into the NGO surface successfully (Fig. 5b).

### Confirmation of binding of DNA-aptamer-NGO composite material to the *P. gingivalis*

FESEM investigation indicates that the untreated *P. gingivalis* cells have rod-like shape (length, 1.99 ± 0.36 μm; diameter, 1.23 ± 0.12 μm) and intact surfaces (Fig. 5c). After exposure to DNA-aptamer-NGO composite for ten min, the mean size of bacteria increased (length, 2.66 ± 0.37 μm; diameter, 1.52 ± 0.22 μm; Fig. 5d) without change in the shape of bacteria which shows evidence for binding of DNA-aptamer-NGO composite material to the *P. gingivalis* as the target.

### The effect of pH on aptamer binding

**Figure 6:**
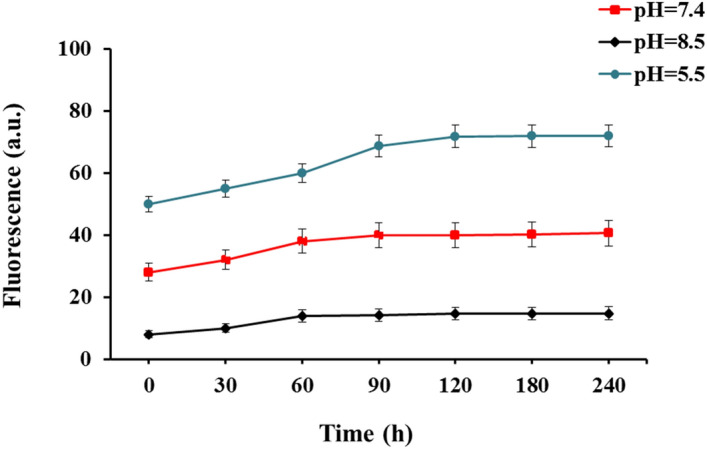
The effect of pH on aptamer binding. pH 8.5 has a positive effect on aptamer binding to the *P. gingivalis*, and pH 5.5 has an adverse effect on aptamer binding to the *P. gingivalis*. The fluorescence intensity of the "aptamer-target complex" at pH 8.5 increases slightly in comparison with pH 7.4 while at pH 5.5 low fluorescence intensity has been observed.

The effect of different pH values on aptamer binding was shown in Fig. 6. Under the alkaline (pH 8.5) and acidic (pH 5.5) conditions, positive and adverse effects on aptamer binding to the *P. gingivalis* were seen respectively during 240 h of incubation at 37 °C in comparison with a neutral condition (pH 7.2). DNA-aptamer-target complex resulted gradually in fluorescence enhancement, reaching a plateau in 90 min. According to the findings within a period of 240 h, about 15% of fluorescence intensity was increased in the alkaline (pH 8.5), while fluorescence intensity was reduced by about 15% in a weak-acid environment (pH 5.5).

### Hemolysis assay

**Figure 7:**
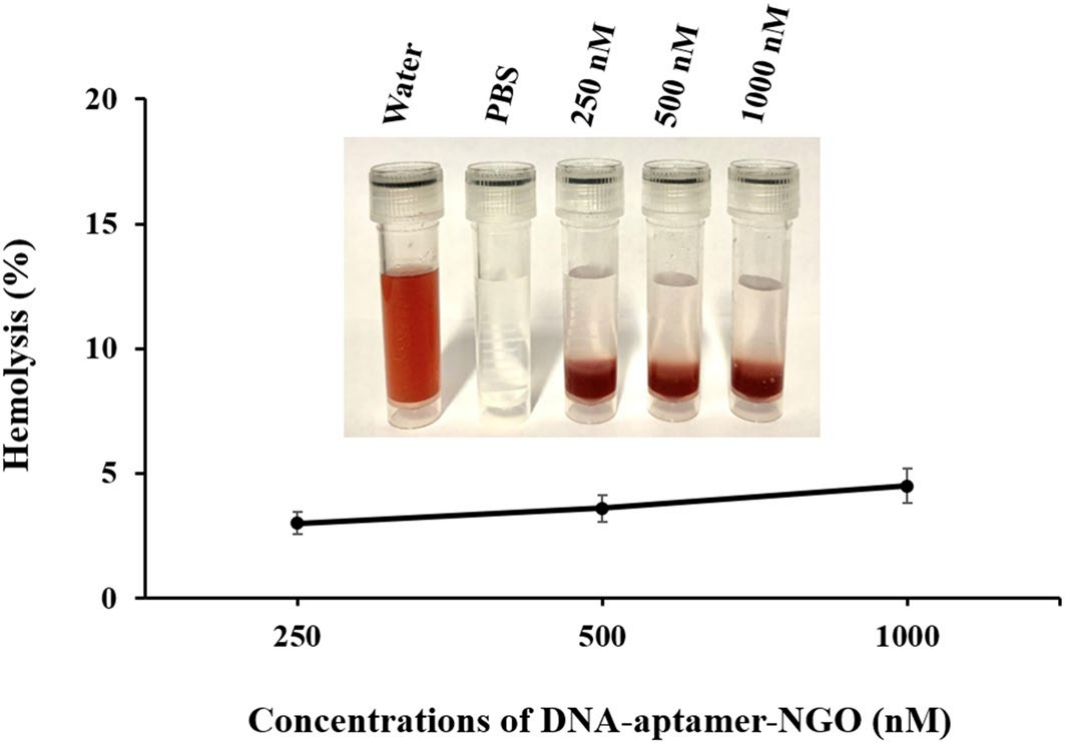
Hemolysis assay of human red blood cells exposed to DNA-aptamer-NGO at the different concentrations (250, 500, and 1000 nM) at a 37 °C water bath for 1 h. The absorbance was analyzed by a UV–Vis spectrophotometer at 540 nm. Distilled water and PBS solution were used as the positive and negative control, respectively.

As shown in Fig. 7, high concentrations of DNA-aptamer-NGO incubated for 24 h with human red blood cells did not exceed 5%. The non-hemolytic character confirms the hemocompatibility of DNA-aptamer-NGO, which was similar to the PBS as a negative control.

### Cytotoxicity effect of DNA-aptamer-NGO on human gingival fibroblast cell

**Figure 8:**
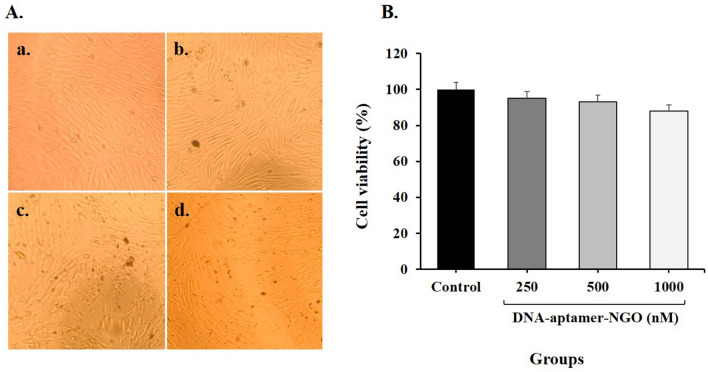
Effects of DNA-aptamer-NGO on cell viability of primary human gingival fibroblast (HGF) cells: (**A**) Inverted light microscope images of treated cells with varying concentrations of DNA-aptamer-NGO (250, 500, and 1000 nM; magnification × 40); (a) Control (Untreated cells), (b) 250 nM, (c) 500 nM, and (d) 1000 nM. (**B**) The mean percentage viability of HGF cells versus concentrations of DNA-aptamer-NGO (250, 500, and 1000 nM) using MTT [3-(4,5-dimethylthiazol-2-yl)-2,5-diphenylte-trazolium bromide] assay.

The MTT assay was performed to evaluate the gingival fibroblast cytotoxicity of DNA-aptamer-NGO. At different concentrations of DNA-aptamer-NGO, the mean percentage of HGF cells viability ranges from 95.2% to 87.6% (Fig. 8). Although the mean percentage of fibroblast viability decreases as the concentrations of DNA-aptamer-NGO (250, 500, and 1000 nM) increases, these reductions were not significant compared to the control group (untreated cells; *P* > 0.05).

### Monitoring of specificity of DNA-aptamer-NGO to *P. gingivalis*

**Figure 9:**
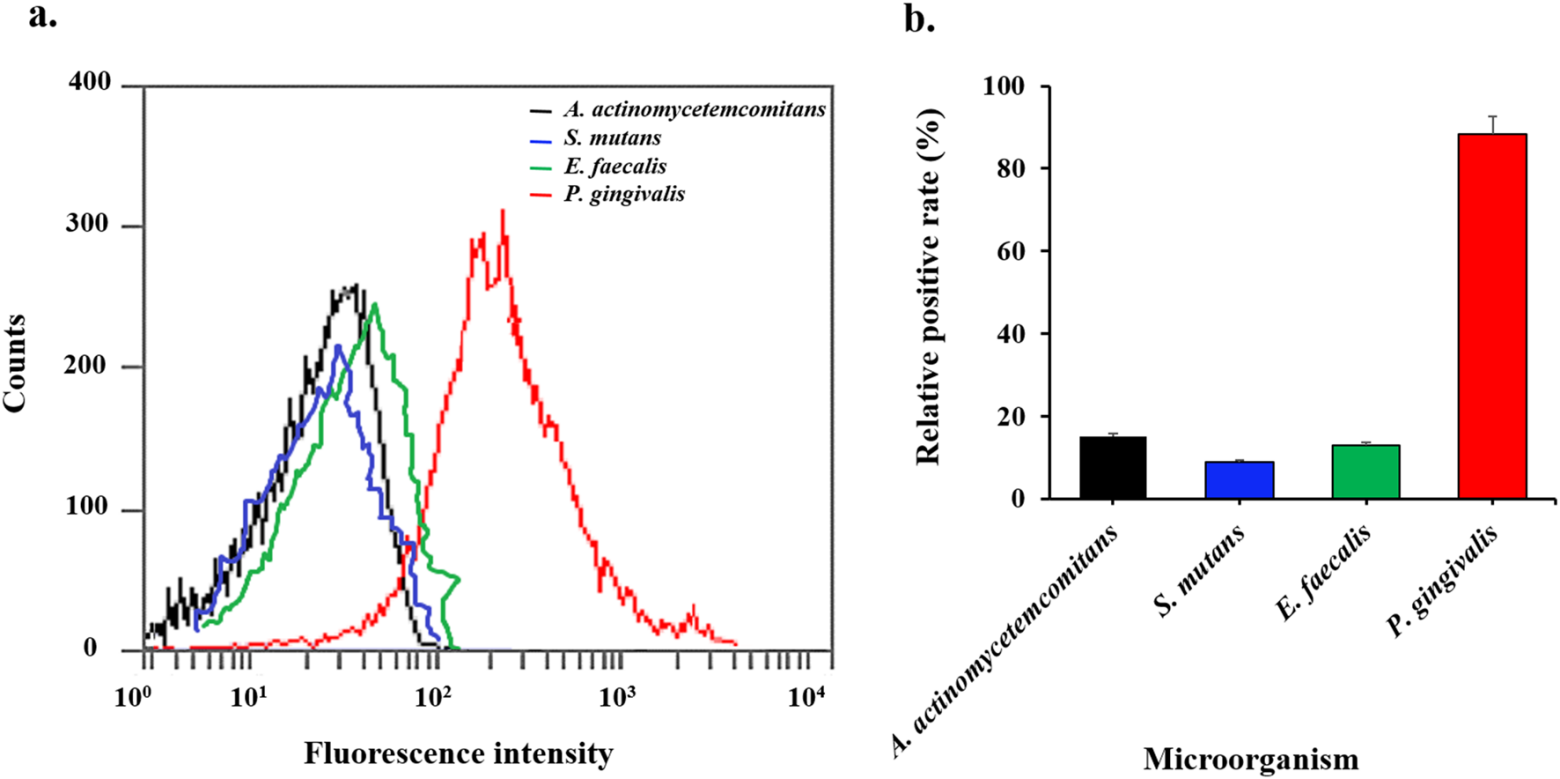
Affinity and selectivity of DNA-aptamer-NGO towards *P. gingivalis*: (**a**) Flow cytometric assay for the binding of the aptamer to different species of bacteria (10^6^ CFU/mL) including *Streptococcus mutans*, *Enterococcus faecalis*, and *Aggregatibacter actinomycetemcomitans* as the non-target cells at 37 °C for 2 h. (**b**) Histogram of the percentage of gated fluorescence intensity of bacteria in terms of relative positive rate**.**

Fluorescently labeled DNA-aptamer-NGO was analyzed against different species of bacteria, including *A. actinomycetemcomitans*, *E. faecalis*, *P. gingivalis*, and *S. mutans*. According to the results, the positive count (fluorescence intensity > 10) of *P. gingivalis* was significantly higher than that of the other bacteria (relative positive rate of 88.3%), indicating that the DNA-aptamer-NGO was of preferential binding affinity to *P. gingivalis*. As shown in Fig. 9, DNA-aptamer-NGO seemed to be of lower affinity to *S. mutans* (relative positive rate of 9.1%) among the other bacteria.

### Determination of MIC and MBC of DNA-aptamer-NGO composite against *P. gingivalis*

In this study, *P. gingivalis* in the presence of DNA-aptamer-NGO composite in doubling dilutions from 31.2 to 0.97 nM displayed visible growth, while at concentrations ≤ 62.5 nM of DNA-aptamer-NGO composite, bacterial growth is not observed. So, the minimum bacteriostatic concentration (MIC) of DNA-aptamer-NGO was 62.5 nM which inhibited the growth of *P. gingivalis* in the broth medium.

Re-culturing (subculturing) broth dilutions at and above the MIC (≤ 62.5 nM) onto BHI agar supplemented with hemin and menadione revealed that DNA-aptamer-NGO at concentrations ≥ 125 nM was prevented the growth of the *P. gingivalis* on the agar plate. So, the minimum bactericidal concentration (MBC) of DNA-aptamer-NGO was 125 nM which implied nonviable *P. gingivalis* on the plate.

### Evaluation of endogenous ROS production

**Figure 10:**
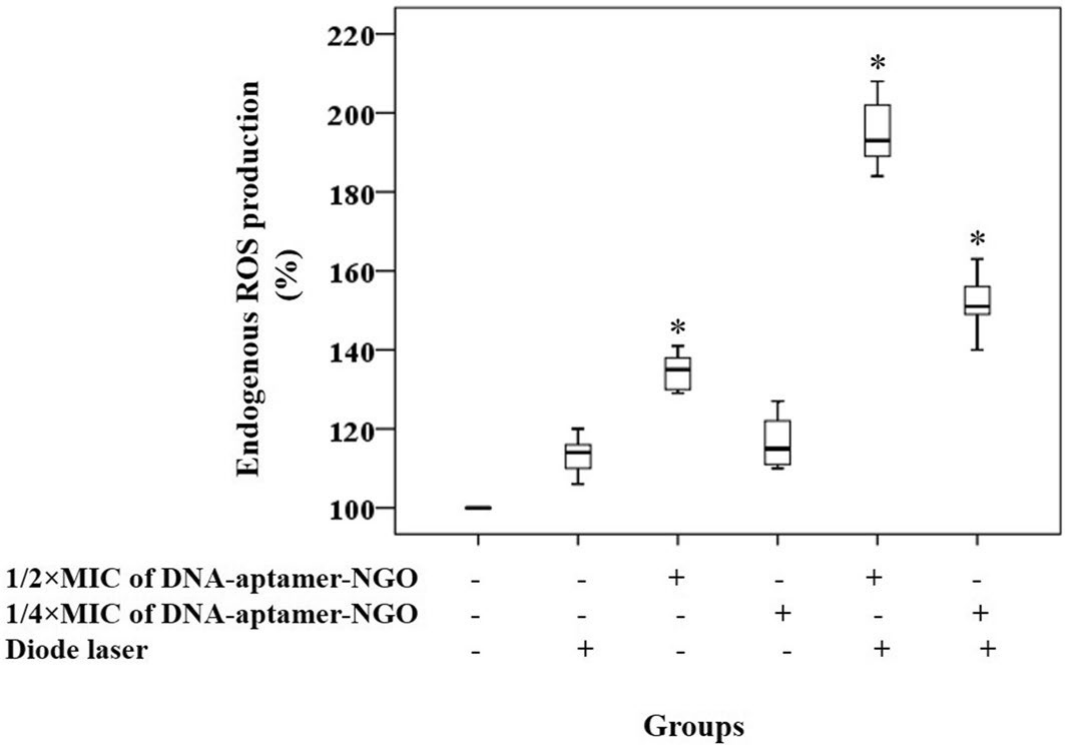
Generation of endogenous ROS. Generated ROS was quantified by fluorescence spectroscopy using 2′,7′-dichlorofluorescein diacetate (H_2_DCF-DA) after treatment of *P. gingivalis* (10^8^ CFU/mL) exposed to DCFH-DA (10 μM) with 1/2 × and 1/4 × MIC of DNA-aptamer-NGO, aPDT (DNA-aptamer-NGO at 1/2 × and 1/4 × MIC plus diode laser), and diode laser. The control group (only bacterial cells) was left untreated. Significant differences according to the control, *P < 0.05.

aPDT groups using 1/2 × and 1/4 × MIC of DNA-aptamer-NGO plus irradiation of diode laser demonstrated the enhanced ROS production by 1.95- and 1.51-fold compared with the control group (untreated *P. gingivalis*), respectively (*P* < 0.05; Fig. 10), which was dose-dependent. Also, the results revealed an increase (1.29-fold) in microbial suspension exposed to 1/2 × MIC of DNA-aptamer-NGO alone, while no significant increase in the DCFH-DA fluorescence was observed in *P. gingivalis* suspension subjected to diode laser alone and 1/4 × MIC of DNA-aptamer-NGO (*P* > 0.05).

### Antimicrobial effect of aPDT based on DNA-aptamer-NGO against *P. gingivalis* in planktonic form

**Figure 11:**
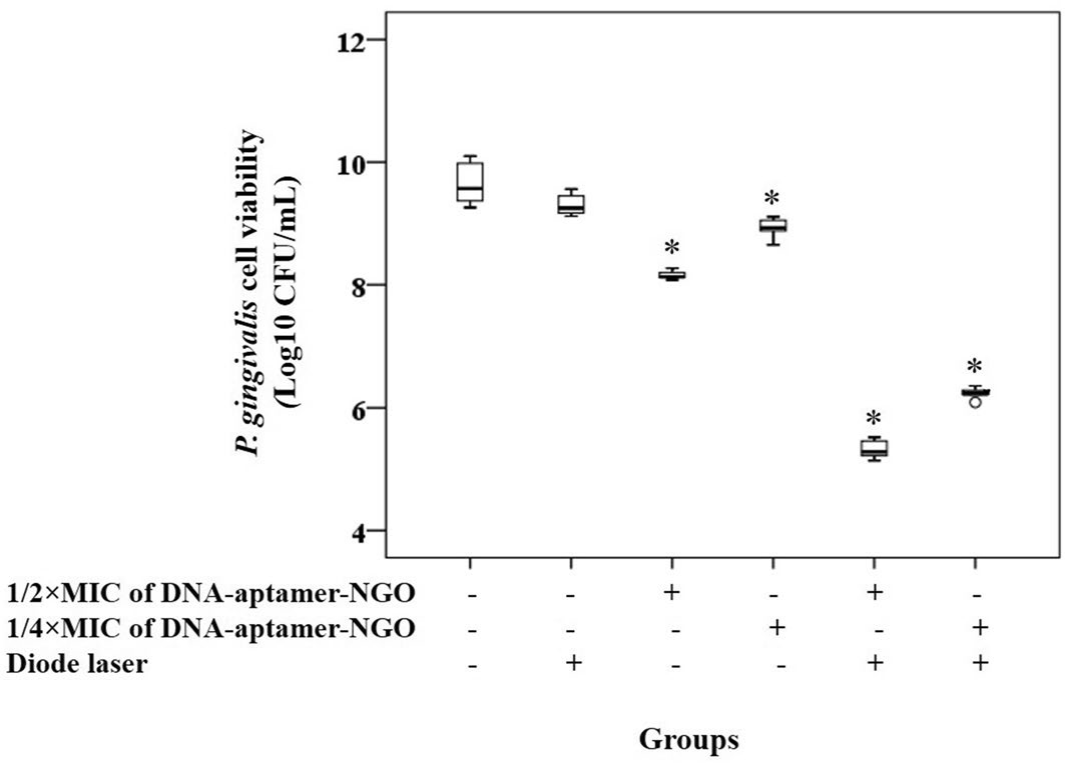
The antimicrobial effect of different treatment groups against *P. gingivalis*. Cell viability of *P. gingivalis* following treatment with 1/2 × and 1/4 × MIC of DNA-aptamer-NGO, aPDT (DNA-aptamer-NGO at 1/2 × and 1/4 × MIC plus diode laser), and diode laser were quantified by colony counting. Significant differences according to the control, *P < 0.05.

Figure 11 shows the Log_10_ CFU/mL reduction rates of *P. gingivalis* after different treatments as compared to untreated *P. gingivalis* suspension. According to the findings, 1/2 × and 1/4 × MIC of DNA-aptamer-NGO alone showed a concentration-dependent decrease of 1.49 and 0.73 Log_10_ for *P. gingivalis*, respectively (*P* < 0.05). Upon irradiation, aPDT with 1/2 × and 1/4 × MIC of DNA-aptamer-NGO led to reductions of 4.33 and 3.42 Log_10_ against *P. gingivalis*, respectively (*P* < 0.05). In contrast, no significant change in *P. gingivalis* viability was observed after irradiation of diode laser light (*P* > 0.05).

### Anti-biofilm effect of aPDT based on DNA-aptamer-NGO against *P. gingivalis*

**Figure 12:**
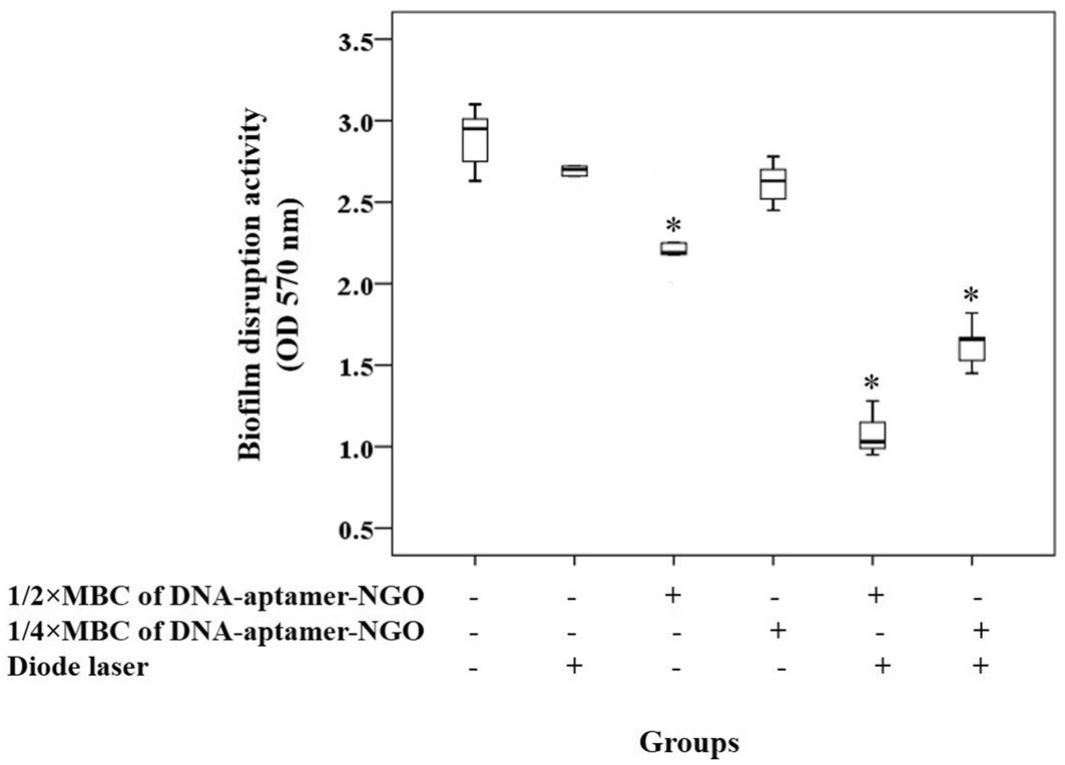
The anti-biofilm effects of different treatment groups against *P. gingivalis*. Biofilm disruption of *P. gingivalis* following treatment with 1/2 × and 1/4 × MIC of DNA-aptamer-NGO, aPDT (DNA-aptamer-NGO at 1/2 × and 1/4 × MIC plus diode laser), and diode laser were determined by crystal violet assay. Significant differences according to the control, *P < 0.05.

Since the microbial biofilms are more resistant to antimicrobial treatment, we used an MBC dose of DNA-aptamer-NGO against *P. gingivalis* in this study. Similar to the effect observed for planktonic cells of *P. gingivalis*, the results of the crystal violet assay in Fig. 12 show that aPDT using 1/2 × and 1/4 × MBC of DNA-aptamer-NGO plus irradiation of the diode laser light (1 min) has a significantly anti-biofilm effect against *P. gingivalis* in comparison with the control group (*P* < 0.05). Although diode laser and 1/4 × MBC of DNA-aptamer-NGO alone were not able to inhibit the biofilm considerably (*P* > 0.05), a significant biofilm degradation was observed in *P. gingivalis* biofilm treated with 1/4 × MBC of DNA-aptamer-NGO alone versus the control group (*P* < 0.05; Fig. 12).

### Assessment of anti-metabolic activity of aPDT based on DNA-aptamer-NGO

**Figure 13:**
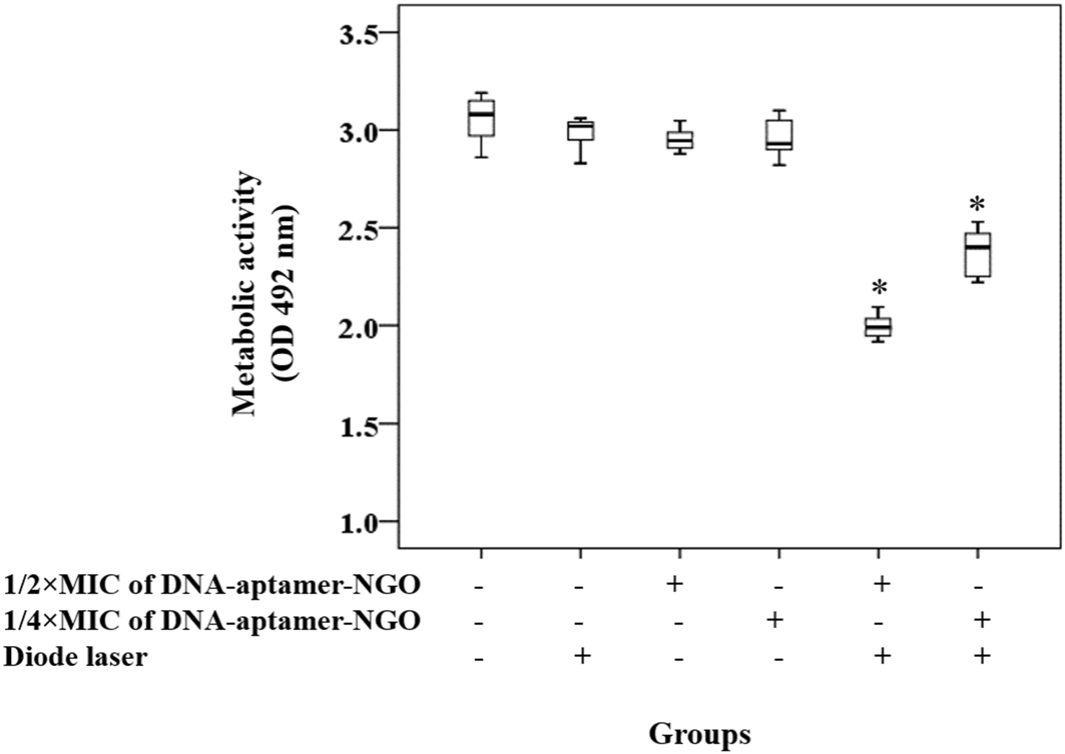
Anti-metabolic effects of different treatment groups against *P. gingivalis.* Metabolic reduction of *P. gingivalis* following treatment with 1/2 × and 1/4 × MIC of DNA-aptamer-NGO, aPDT (DNA-aptamer-NGO at 1/2 × and 1/4 × MIC plus diode laser), and diode laser were determined using the XTT (2,3-bis [2-methyloxy-4-nitro-5-sulfophenyl]-2H-tetrazolium-5-carboxanilide) reduction assay. Significant differences according to the control, *P < 0.05**.**

The aPDT results showed that the metabolic activity of *P. gingivalis* was more sensitive to 1/2 × MIC of DNA-aptamer-NGO, which resulted in 40.3% inactivation compared to 1/4 × MBC of DNA-aptamer-NGO, where only 22.2% inactivation was observed after similar light irradiation (both *P* < 0.05; Fig. 13). In contrast, there was no significant anti-metabolic activity was observed in *P. gingivalis* treated with 1/2 × and 1/4 × MIC of DNA-aptamer-NGO and diode laser alone (all less than 4.0%; *P* > 0.05).

### The apoptotic effects of aPDT based on DNA-aptamer-NGO

**Figure 14:**
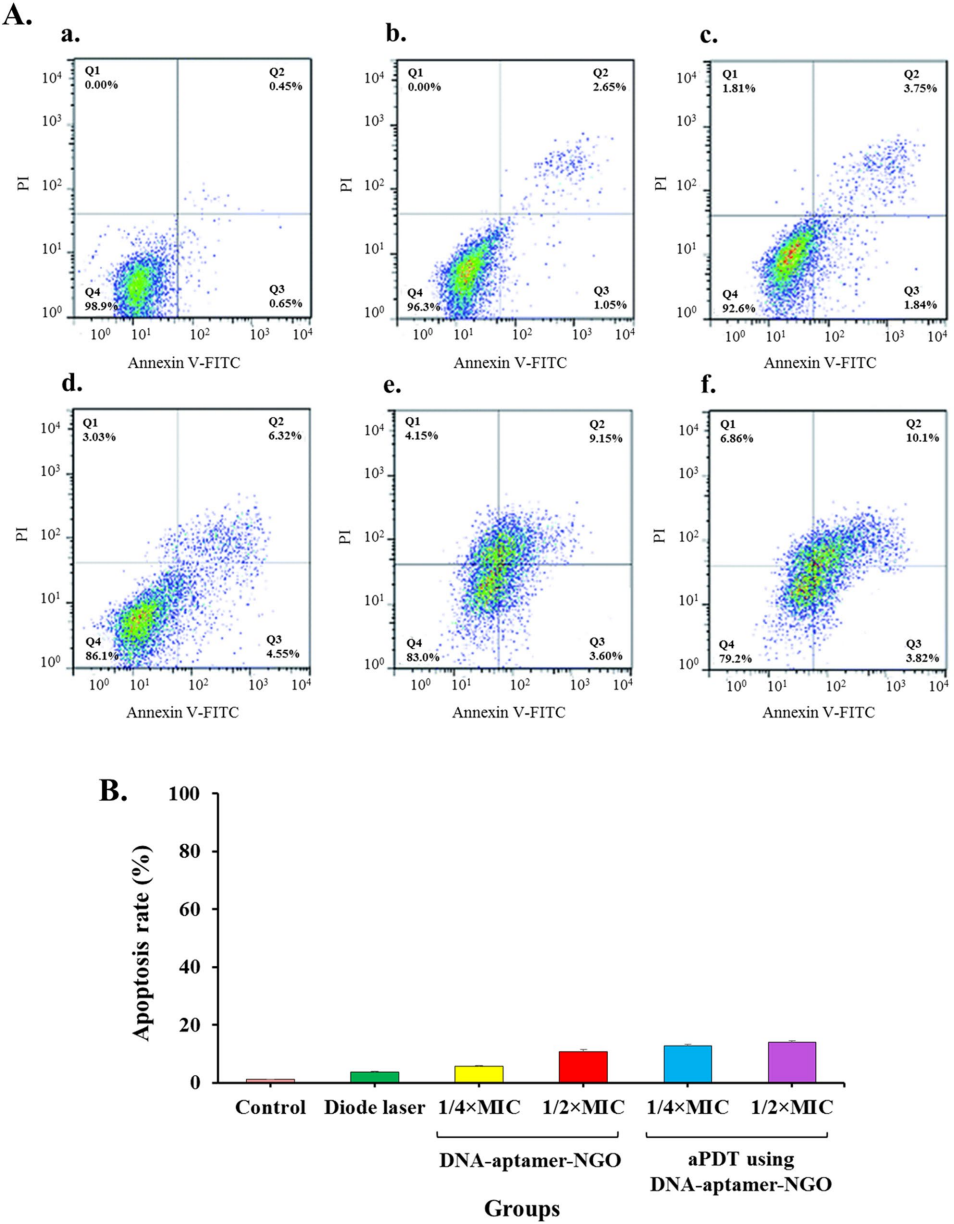
Determination of the apoptotic effects in treated human gingival fibroblast cells with different treatment groups: (**A**) The apoptotic ratio was assessed by flow cytometry with Annexin-V/-FITC/PI staining in human gingival fibroblast cells: (a) control (untreated cells), (b) diode laser, (c) 1/4 × MIC of DNA-aptamer-NGO, (d) 1/2 × MIC of DNA-aptamer-NGO, (e) aPDT using 1/4 × MIC of DNA-aptamer-NGO plus diode laser, and f. aPDT using 1/2 × MIC of DNA-aptamer-NGO plus diode laser; (**B**) Percent of cell viability at 540 nm.

The percentage of apoptotic cells was quantified by flow cytometric analysis. As shown in Fig. 14a, compared with the control group (Annexin V [0.65%] and PI [0.00%]), the number of apoptotic cells only slightly increased in the diode laser alone group, and the 1/2 × and 1/4 × MIC of DNA-aptamer-NGO without light group. However, the number of apoptotic cells increased in the aPDT groups. According to the results, the percentage of apoptosis cells treated with DNA-aptamer-NGO at 1/2 × and 1/4 × MIC plus diode laser were 13.9% and 12.7%, respectively (Fig. 14b).

### Evaluation of genes expression

**Figure 15:**
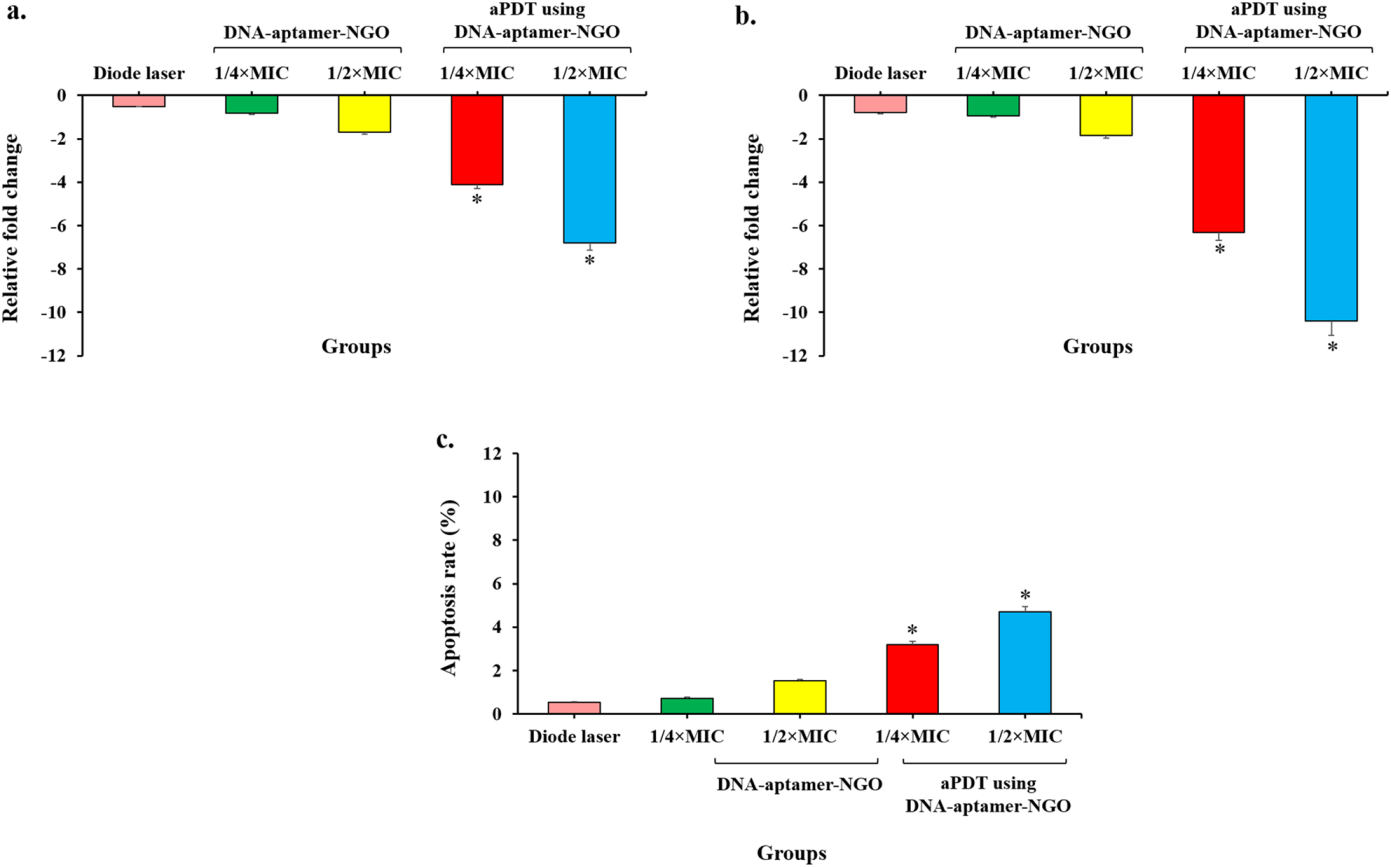
mRNA expression levels of *P. gingivalis* following different treatment groups*.* The relative fold change in mRNA expression levels of *P. gingivalis* following treatment with 1/2 × and 1/4 × MIC of DNA-aptamer-NGO, aPDT (DNA-aptamer-NGO at 1/2 × and 1/4 × MIC plus diode laser), and diode laser were determined using the RT-qPCR. Significant differences according to the control, *P < 0.05.

The expression level of *rgpA* gene significantly decreased after exposure to aPDT groups compared with the control group (untreated *P. gingivalis*; *P* < 0.05; Fig. 15). As shown in Fig. 15, the expression of *rgpA* gene was downregulated to approximately 6.8- and 4.1-fold following aPDT using 1/2 × and 1/4 × MIC of DNA-aptamer-NGO, respectively (*P* < 0.05). DNA-aptamer-NGO plus diode laser reduced the expression of *fimA* gene significantly. The greatest reduction was observed for aPDT using 1/2 × MIC of DNA-aptamer-NGO, which was approximately 10.4-fold (*P* < 0.05). According to the findings, the profile of *oxyR* gene expression was enhanced in *P. gingivalis* cells, with the greatest increase seen for aPDT using 1/2 × and 1/4 × MIC of DNA-aptamer-NGO, which were 4.7- and 3.2-fold, respectively (*P* < 0.05). On the other hand, there was no significant difference in gene expression when the *P. gingivalis* were treated with DNA-aptamer-NGO and diode laser alone (*P* > 0.05).

## Discussion

In the current study, targeted identification of *P. gingivalis* as the most important microorganism involved in periodontitis using DNA-aptamer-NGO was conducted, and the antimicrobial potential of aPDT using DNA-aptamer-NGO against *P. gingivalis* was evaluated. Periodontal diseases ensue following the establishment of polymicrobial subgingival biofilms, and *P. gingivalis* is the foremost among these microbial pathogens. *P. gingivalis* can bind to epithelial cells, erythrocytes, fibroblasts, and components of the extracellular matrix via fimbriae and proteolytic enzymes^47^. The systematic use of antibiotics in the treatment of periodontitis is limited due to the need for high doses to achieve the proper concentration of the drug in the gingival sulcus, the rapid onset of bacterial resistance to antibiotics, and the side effects of drugs. In addition, due to the advanced structure and function of the subgingival biofilm, antibiotics may be ineffective or inactivated^48^.

Recently, single antimicrobial agents-carrying drug delivery systems have been introduced to prevent systemic side effects of antibiotics and drug resistance due to oral administration of antibiotics in the treatment of periodontitis^48^. The aptamer can play the role of a specific carrier of antimicrobial agent and increase the effectiveness of treatment, prevent the removal of the beneficial and effective microbiome in health, the development and spread of antibiotic resistance, and the side effects of systemic administration of drugs^49,50^.

Identification of the aptamer candidate is feasible by designing whole-cell SELEX with the addition of a label. Inhere, DNA-aptamer was labeled with FAM and the binding of labeled DNA-aptamer to NGO was carried out based on the modified Lin et al. method^30^. The principle of the detection of *P. gingivalis* using FAM-modified aptamer is based on the continuous process of quenching the fluorescence of NGO and returning fluorescence in the presence of *P. gingivalis*. In the absence of *P. gingivalis*, FAM-aptamer is adsorbed onto the surface of NGO via weak binding force by π-π stacking, and NGO quenches the fluorescence of FAM. In the presence of *P. gingivalis*, the aptamers fall off from the NGO surface and bind to the *P. gingivalis*, causing fluorescence restoration.

Tan et al.^51^ have shown that the fluorescence quenching of FAM-aptamer occurs rapidly following its binding to the GO surface (i.e. the formation of FAM-aptamer-GO composite) and tends to decrease significantly after 2 min. In contrast, upon the addition of CEM (human acute leukemic lymphoblast cell lines), the CEM-FAM-aptamer was formed slowly and the release of the FAM-aptamer from the GO surface was slower and took more than 30 min. So, the fluorescence quenching by the GO can be gradually restored. Accordingly, during the period when the FAM-aptamer-GO composite is attached to the target and before the GO is released from the target-FAM-aptamer-GO complex, if the light is irradiated on the complex, the effects of aPDT can appear.

In this study, DNA aptamers are selected and desorbed from NGO surfaces in the presence of target molecules at close to the neutral pH (pH 7.4 and high ionic strength). A slight increase in the pH (i.e., pH 8.5 and high ionic strength) has been shown to have a positive effect on aptamer binding to the target, and lowering the pH (pH 5.5 and high ionic strength) has had an adverse effect on aptamer binding to the target. The fluorescence intensity of the "aptamer-target complex" at pH 8.5 has been shown to increase slightly in comparison with pH 7.4 while at pH 5.5 no obvious fluorescence intensity has been observed. Huang et al.^32^ revealed that the binding equilibrium can be shifted by simply tuning the solution pH. At lower pH (pH 3.5), the aptamer-GO binding is enhanced while the aptamer-target binding is weakened, making this system a regenerable biosensor without covalent conjugation. The acidification of the “target-FAM-aptamer-GO complex” by incubating with 500 mM, pH 3.5, leads to regeneration. At this pH, the FAM-aptamer releases the bound target and re-adsorb onto the GO surface and leading to the formation of the FAM-aptamer-NGO composite. The fact that the pH 3.5 sample showed a much lower fluorescence supported that a low pH was crucial for shuttling the FAM-aptamer back to the GO surface.

On the evaluation of the biosafety aspects of nanomaterials for in vivo, hemocompatibility and cytotoxicity effects should be assessed. In this study, the hemolysis assay was performed to test the hemolytic activity of DNA-aptamer-NGO by studying their effect on erythrocytes. As the results have shown, DNA-aptamer-NGO was compatible with blood cells and the medium remained colorless due to the coagulation property of blood cells. Also, the percentage of cytotoxicity of DNA-aptamer-NGO at different concentrations ranged from 4.8% to 12.4%. No significant difference was observed compared to the control group.

One of the benefits of using an aptamer-based drug delivery system in the treatment of periodontitis is the targeted therapy in the treatment of periodontitis by binding specifically to the organism which aptamer is designed for it. Thus, the microflora effective in creating periodontal health is not destroyed and the possibility of their presence and, as a result, the possibility of returning health to the periodontium is raised. According to the results of this study, DNA-aptamer-NGO demonstrated a preferential binding affinity for *P. gingivalis* over the other bacteria tested.

Aptamers obtained through the SELEX strategy can bind to target sites. Numerous studies have detailed the selection of aptamers against different microbial species in recent years^52–58^. Wang et al.^53^, Davydova et al.^54^, and Soundy and Day^55^ provided several aptamers as specific bio-recognition molecules for the rapid detection, early diagnosis, and therapeutic targeting of *Pseudomonas aeruginosa*. The findings of Bayraç et al.^56^ suggest that DNA-aptamers may be potential probes for further research into the proteins responsible for *Streptococcus pneumoniae* biofilm. Hu et al.^57^ developed DNA-aptamers for the detection of *Bifidobacterium breve*. They reported the developed colorimetric bioassay based on the aptamer BB16-11f. is a promising method for the detection of *B. breve*. In another study, Saad et al.^58^ provided a cell-SELEX strategy in identifying two aptamers binding to *Legionella pneumophila* in a water system with high affinity.

To the best of our knowledge, no study has been conducted to investigate the antimicrobial application of DNA-aptamer-NGO during aPDT. In the present study, after confirming the binding of DNA-aptamer-NGO to *P. gingivalis*, its antimicrobial properties were evaluated in both planktonic and biofilm phases. The pH-responsive DNA-aptamer-NGO-complex could facilitate the accumulation of DNA-aptamer-NGO in bacterial cells and hence greatly improve the effect of aPDT against *P. gingivalis*. According to the findings, non-toxic and non-hemolytic DNA-aptamer-NGO with diode laser irradiation reduced *P. gingivalis* Log_10_ CFU/mL. Similar to the effect observed for planktonic cells of *P. gingivalis*, aPDT using DNA-aptamer-NGO had significantly anti-biofilm effect against *P. gingivalis*. Therefore, not only aptamer specifically identify *P. gingivalis* as the target bacterium, but it also played an antimicrobial role in the degradation of *P. gingivalis* biofilm in aPDT process by an NGO-carrying drug delivery system. In addition, the metabolic activity of *P. gingivalis* was more sensitive to aPDT based on DNA-aptamer-NGO.

The previous studies confirmed the application of aptamers in interference of cell apoptosis^59–63^. It has been proven that aptamers regulate the extrinsic (by activating specific sensors or genes to induce or suppress apoptosis) and intrinsic (by trigging and transducing the apoptotic signals from the extracellular medium to the intracellular medium after targeting death receptors) pathways of apoptotic in the cancer cells^60^. In this study, the apoptotic effect of aPDT using DNA-aptamer-NGO was determined. As the results displayed, although the number of apoptotic cells increased in aPDT groups (1/2 × and 1/4 × MIC plus diode) through ROS generation, these inductions of apoptosis were not significant. Moreover, the anti-virulence activity of DNA-aptamer-NGO-mediated aPDT showed that the expression level of genes (*rgpA* and *fimA*) involved in bacterial biofilm formation decreased significantly after exposure to 1/2 × and 1/4 × MIC of DNA-aptamer-NGO, while the expression level of *oxyR* as a gene involved in response to oxidative stress increased considerably; the former reduces the pathogenesis and the latter increases the possibility of elimination of microorganisms.

## Conclusion

Based on the results, DNA-aptamer-NGO can detect *P. gingivalis* in microbial complexes with the highest binding affinity. An increase in the antimicrobial, anti-biofilm, and anti-metabolic, as well as, changes in the expression level of genes involved in pathogenicity followed by the excessive intracellular ROS generation induced by aPDT in the presence of DNA-aptamer-NGO were the main responsible for the significant elimination of *P. gingivalis*. In conclusion, our results suggest that DNA-aptamer-NGO as a bio-theragnostic element can be used as a promising candidate to detect *P. gingivalis* in periopathogenic complex in real-time and in situ.

## Data Availability

All datasets supporting the conclusions of this article are included within the article.
